# Seeing the Forest through the Trees: Considering Roost-Site Selection at Multiple Spatial Scales

**DOI:** 10.1371/journal.pone.0150011

**Published:** 2016-03-30

**Authors:** David S. Jachowski, Christopher T. Rota, Christopher A. Dobony, W. Mark Ford, John W. Edwards

**Affiliations:** 1 Department of Forestry and Environmental Conservation, Clemson University, 258 Lehotsky Hall, Clemson, South Carolina, 29634–0310, United States of America; 2 School of Life Science, University of KwaZulu-Natal, Durban, South Africa; 3 Department of Fisheries and Wildlife Sciences, University of Missouri, 302 Natural Resources Building, Columbia, Missouri, 65201, United States of America; 4 Division of Forestry and Natural Resources, West Virginia University, Box 6125, Morgantown, West Virginia, 26506, United States of America; 5 Fort Drum Military Installation, Natural Resources Branch, Fort Drum Military Installation, Natural Resources Branch, 85 First Street West, IMNE-DRM-PWE, Fort Drum, New York, 13602, United States of America; 6 U.S. Geological Survey, Virginia Cooperative Fish and Wildlife Research Unit, Department of Fisheries and Wildlife Conservation, 106 Cheatham Hall, Virginia Polytechnic Institute and State University, Blacksburg, Virginia, 24061, United States of America; Università degli Studi di Napoli Federico II, ITALY

## Abstract

Conservation of bat species is one of the most daunting wildlife conservation challenges in North America, requiring detailed knowledge about their ecology to guide conservation efforts. Outside of the hibernating season, bats in temperate forest environments spend their diurnal time in day-roosts. In addition to simple shelter, summer roost availability is as critical as maternity sites and maintaining social group contact. To date, a major focus of bat conservation has concentrated on conserving individual roost sites, with comparatively less focus on the role that broader habitat conditions contribute towards roost-site selection. We evaluated roost-site selection by a northern population of federally-endangered Indiana bats (*Myotis sodalis*) at Fort Drum Military Installation in New York, USA at three different spatial scales: landscape, forest stand, and individual tree level. During 2007–2011, we radiotracked 33 Indiana bats (10 males, 23 females) and located 348 roosting events in 116 unique roost trees. At the landscape scale, bat roost-site selection was positively associated with northern mixed forest, increased slope, and greater distance from human development. At the stand scale, we observed subtle differences in roost site selection based on sex and season, but roost selection was generally positively associated with larger stands with a higher basal area, larger tree diameter, and a greater sugar maple (*Acer saccharum*) component. We observed no distinct trends of roosts being near high-quality foraging areas of water and forest edges. At the tree scale, roosts were typically in American elm (*Ulmus americana*) or sugar maple of large diameter (>30 cm) of moderate decay with loose bark. Collectively, our results highlight the importance of considering day roost needs simultaneously across multiple spatial scales. Size and decay class of individual roosts are key ecological attributes for the Indiana bat, however, larger-scale stand structural components that are products of past and current land use interacting with environmental aspects such as landform also are important factors influencing roost-tree selection patterns.

## Introduction

North American bat species face challenges from legacy and current habitat degradation, winter hibernacula disturbance, wind energy impacts on migratory species, and the unprecedented negative effects of White-nose Syndrome on cave-hibernating bats [1,2,3]. In response, managers need to address current and historic threats (e.g., [4]) as well as direct conservation efforts that enhance recruitment and survival of remnant bat populations [5]. While recruitment and survival are often influenced by a suite of species-specific factors, in general, providing habitat conditions ideal for enhancing survival and recruitment during the summer maternity season is widely viewed as one of the most critical components to the conservation and restoration of bat populations [6].

Outside of the winter hibernating season, bats in temperate environments spend their diurnal time in day-roosts, making conditions associated with roosting a key component to bat conservation [7]. Providing access to high -quality day roost sites during the summer maternity season allows bats to conserve energy reserves through thermoregulation and provides optimal conditions for growth of offspring, while also protecting bats from weather and predation [8]. Previous studies of many forest bat species have shown that roosts often are located in trees or snags of large diameter relative to the surrounding forest stand where a high degree of intra-stand decadence is present and solar radiation is high, usually in close proximity to foraging habitat [9,10]. However, considerable variation does exist, producing results that often are specific to a given bat species, population or region [11]. Further, most studies of bat roost ecology focus at a single scale, either the characteristics of the roost tree itself [12,13] or forest stand or landscape-level characteristics [14], with few attempts to assess factors effecting roost site selection across these multiple spatial scales. Thus, an understanding of bat roost-site selection not only should take into account the attributes of individual roost trees, but also how larger forest stand or landscape scale attributes influence roost selection [10,15].

Understanding roost-site selection is particularly important for species of high conservation concern such as the federally-endangered Indiana bat (*Myotis sodalis*). These data are needed to help guide forest and other land management decisions where the bat is present [11,16,17]. Indiana bats were first listed as endangered in 1966 under the United States Endangered Species Preservation Act (later the Endangered Species Act), and despite showing modest population increases through the mid-2000’s, populations in the northeast and mid-Atlantic regions have been decimated over the past decade following exposure to White-nose Syndrome [18,19,20]. Further, Indiana bat conservation is particularly important in their northern distribution because models of predicted climate change indicate that these regions provide key winter hibernacula sites [21]. To facilitate Indiana bat recovery, forest management that conserves and enhances the availability of adequate roost trees has been identified as a key, actionable priority [22]. Many factors, operating at different spatial scales, are hypothesized to influence Indiana bat roost-site selection [16]. Indiana bats exhibit considerable plasticity in selection of roost tree species, with day-roosts occurring in a wide diversity of mixed-deciduous forests [16]. However, individual roost trees or snags usually are characterized by high availability of exfoliating bark for roost crevices [17,23,24] with high solar exposure that increases roost temperature and, consequentially, fetal development [25]. Therefore, at the northern extent of Indiana bat distribution where temperatures are typically cooler and thermoregulatory needs are greatest, understanding patterns in roost-site selection is particularly important for successful conservation.

**Fig 1:**
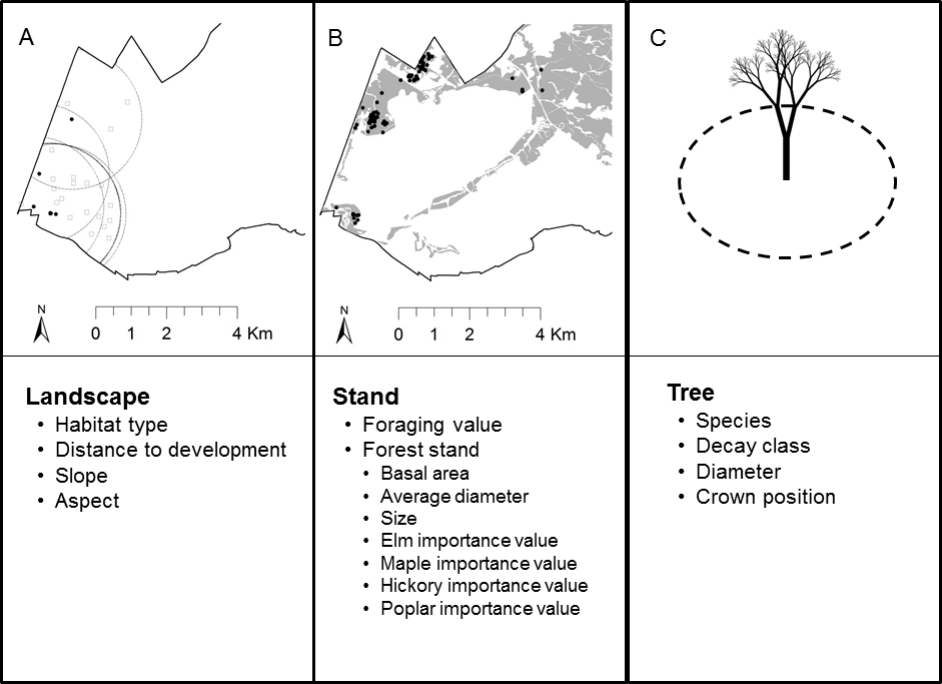
Diagram illustrating 3 scales of investigation (landscape, stand, and roost tree) in Indiana bat roost-site selection at Fort Drum Military Installation, New York, USA between 2007–2011 and habitat covariates evaluated at each scale. At the landscape and stand scales, we identified used sites that were paired with 5 available points (square points) for resource selection analysis. Panel A illustrates the location of used roost sites for Indiana bat 150–282 (solid points) along with a 1976-m radius buffer (dashed line) used to define availability surrounding each roost site. The Fort Drum Installation boundary is depicted by solid black line. Panel B illustrates the location of all roost sites (solid points) and forest stands for which stand attribute data were collected are in gray. Roost-site selection at the tree scale (Panel C) was evaluated based on all available trees within a 0.0405-ha plot centered on the used roost tree.

In this study, we evaluated summer roost-site selection of Indiana bats at Fort Drum Military Installation in northwestern New York, USA, at three spatial scales: landscape, forest stand, and individual tree (Fig 1). At the landscape scale, we hypothesized that roost-site selection would be influenced by landscape-scale topographic features such as slope and aspect, habitat conditions such as forest type and distance to human development, and proximity to foraging areas [16,25]. At the forest-stand scale, we hypothesized that Indiana bat day-roost-site selection would be influenced by proximity to high-quality foraging areas and forest intra-stand characteristics such as forest community composition, area and age class, and stand condition [26,27]. At the roost-tree scale, we hypothesized that Indiana bats would select specific roost trees based on attributes such as tree species, bole size (diameter at breast height), and decay class [23,28]. Identification of factors describing roost selection at multiple scales would help inform forest management decisions aimed at providing high-quality roosting habitat for this species.

## Methods

### Study sites

We conducted our study within and adjacent to Fort Drum Military Installation (Fort Drum), a 43,000+ ha United States Army facility in Jefferson and Lewis counties, New York, USA. Fort Drum was located in the northcentral portion of the state at the intersection of the Tug Hill Plateau, the St. Lawrence/Great Lakes Lowlands and the foothills of the Adirondack Mountains. Limestone “Karst” formations in the Niagara Escarpment, 10–15 km west of Fort Drum, contained caves where bats hibernate during the winter. Topography was rolling with some incised watercourses along the Black and Indian River drainages. Elevations ranged from 125–278 m. Approximately 70% of Fort Drum was forested. Mature forests were mainly northern mixed hardwood associations of sugar maple (*Acer saccharum*), red maple (*A*. *rubrum*), American beech (*Fagus grandifolia*), white ash (*Fraxinus americana*), black cherry (*Prunus serotina*), American elm (*Ulmus americana*) and basswood (*Tilia americana*), mixed with a conifer component of white pine (*Pinus strobus*) and eastern hemlock (*Tsuga canadensis*). Wetter areas contained primarily eastern hemlock, red maple, eastern cottonwood (*Populus deltoides*), black willow (*Salix nigra*) and silver maple (***A*. *saccharinum)*. *Drier sites contained northern red oak (Quercus rubra)*, *white pine*, *red pine (P*. *resinosa) and planted Scots pine (P*. *sylvestris)*.** In addition to developed areas within the installation’s 4500 ha cantonment area and airfield, open habitat types that exhibited a variety of early successional forest growth stages occurred throughout areas maintained for training, such as drop-zones, firing ranges, maneuver areas, and forest regeneration sites (often dominated by quaking aspen; *Populus tremuloides*, and gray birch *Betula populifolia*). Small lakes, beaver (*Castor canadensis*) ponds, and open wet meadows covered approximately 20% of the landscape [18]. Lands adjacent to Fort Drum included similar elevations and forest associations, but many forested areas around the installation were more highly fragmented and interspersed with larger overall areas of agricultural row crops, pasture land and urban development [29].

### Field monitoring

We captured Indiana bats opportunistically during August and September of 2007–2011 using mist nets (38 mm mesh, Avinet, Inc., Dryden, NY) over water, forest roads and trails, along forest-field edges and forest canopy gaps. Mist nets were opened for up to 5 hours following sunset. Upon capturing a bat, we determined species, sex, age (juvenile or adult), weight, forearm length and reproductive condition (i.e., non-reproductive or reproductive male, or non-reproductive, pregnant, or lactating female). We used Skin Bond® (Smith and Nephew, Largo, FL) surgical cement to affix a 0.35-g radiotransmitter (Blackburn or Holohil Transmitters, Nacogdoches, TX and Carp, Ontario, Canada, respectively) between the scapulae of each captured Indiana bat. We also placed a uniquely identifiable metallic split-ring band (Porzana Ltd, Icklesham, UK) on the sex-appropriate forearm of each bat. Bat capture and handling protocols were approved by the Animal Care and Use Committee of West Virginia University (Protocol No. 08–0504), followed the guidelines of the American Society of Mammalogists [30], and were in accordance with requirements of a New York State Department of Environmental Conservation Endangered/Threatened Species Scientific Collecting Permit. To minimize radiotransmitter effects on bat movement, the radiotransmitter to body mass ratio was limited to no more than 5% for tagged bats [31].

We radiotracked bats to their day-roost tree (hereafter day-roost [10,17]) during summer (May 15-August 15) and fall (August 15 –October 15) between 2007 and 2011. We recorded day-roost location and tree characteristics, including species, decay class, diameter at breast height (cm), and crown position. Crown position of each tree was determined to be either dominant (*D*), co-dominant (*C*), intermediate (*I*), or suppressed (*S*) [17]. Decay class was categorized on a scale of 1 to 8, where 1 represented a living tree; 2 a live, but declining or dying tree; 3 a recently dead tree; 4 a dead tree with loose bark; 5 a dead standing snag with limited or no bark; 6 a broken topped tree with or without residual bark; and 7 and 8 represented trees in advanced state of decay to the point of becoming a residual stump or prostrate coarse woody debris [32,33,34].

We paired used day-roosts with randomly available day-roosts to evaluate which characteristics were associated with day-roost selection. We selected randomly available day-roosts at the landscape and stand scales by buffering each used day-roost by the average maximum distance traveled during a night among Indiana bats included in this study (see [29]), which was 1976 m, and selecting 5 random points within the buffer (Fig 1). At the stand scale, some used day-roosts (<10%) occurred outside of the area where stand-scale data existed from Fort Drum’s forest inventory program, and were censored from our analyses (Fig 1). We assumed all trees within 11.4 m (0.0405 ha) of the used day-roost were available for use at the tree scale [35].

### Variable selection

#### Landscape scale

We hypothesized that landscape-scale roost-site selection occurred as a function of proximity to hardwood forests, development disturbance, and topographic features (Fig 1). In contrast to portions of the midwestern United States where Indiana bats primarily roost in hydric forests, Indiana bats in the East often roost in hardwood trees on mid-to-upper elevation sites [17,36,37]. Therefore, we hypothesized that Indiana bats would select roost sites in northern mixed forests as opposed to riparian or swamp forests. Indiana bats are known to roost near suburban development [38,39,40], but a finer-scale relationship of their proximity to buildings and roadways (human development) and likely associated disturbances within protected areas remains unknown [16]. Therefore, we hypothesized a positive effect of increasing distance to development on bat roost- site selection at Fort Drum. Thermoregulatory properties of roost sites are particularly important for day-roost sites for bats, particularly at the limits of their northern distribution [41,42]. In addition to canopy cover, topographic features of both slope and aspect can influence solar exposure and thermal features of a roost and have been found to be important factors in Indiana bat space use, particularly in northern temperate forests and in the Appalachian Mountains [17,42]. Therefore, we hypothesized that Indiana bats would select roost sites on or near south-facing slopes due to warmer micro-site temperatures [17].

We used the Northeast Terrestrial Habitat Classification System land cover maps to categorize local Fort Drum habitat conditions into either unforested, central hardwood swamp, northern mixed forest, or developed [43]. Unforested areas were not completely devoid of trees but rather contained <20% of landcover occupied by trees, and typically consisted of agricultural, open water, shrub marsh, or emergent marsh habitat classifications. Therefore, these areas could contain potential bat day-roosting habitat. We recorded habitat classification at used and available day-roosts and also measured the shortest distance between day-roosts and developed areas. We obtained slope and aspect measures from U.S, Geological Survey digital elevation models for the study area from the New York State GIS Clearinghouse (www.gis.ny.gov, accessed June 2013). Because aspect is based on circular degrees, we transformed angular data to radians and centered our metric on the south aspect using cos(aspect) + 1, which yielded values that ranged from 0 (at 180°, or S) to 2 (at 0 and 360°, or N) [26].

#### Stand scale

At the scale of the forest stand (i.e., a contiguous group of trees sufficiently uniform in species composition, age class, and condition to be considered a distinguishable unit [30]), Indiana bats tend to select stands containing large diameter trees with exfoliating or sloughing bark under which they can roost, or stands containing a high proportion of dead or dying trees that provide plates of detaching bark and/or roost cavities [16,27,37,44]. Therefore, we hypothesized that Indiana bats would select for roost sites within stands with high relative basal areas and containing trees with larger average diameters at breast height (DBH) (Fig 1). Further, we hypothesized that stands of greater area would be selected, given their higher likelihood of containing suitable roost trees, and lower risk of disturbance [16]. Within stands, we hypothesized that stand composition and availability of certain large-diameter tree species would influence roost-site selection. For example, based on previous research, we hypothesized that stands containing a large proportion of American elm [44], sugar and red maples [45], bitternut hickory (*Carya cordiformis*) [25], and eastern cottonwood [16] would be selected by Indiana bats for day-roosts. Accordingly, we calculated the Importance Value [46] of each of these four tree species/species groupings within each stand using the formula:

$$\text{Importance Value}=\frac{\#\;\text{stems species x}}{\text{total stems}}+\frac{\text{basal area species x}}{\text{total basal area}}$$

whereby the number of stems was determined as the number of species *x* stems >2.54 cm within each 10-factor prism plot extrapolated out to the larger stand area [34]. Similarly, basal area of each species was estimated within plots using standard silvicultural measurements and formulae [34].

In addition to forest stand attributes, we predicted that roost-site selection at the stand scale was also likely influenced by proximity to high-quality foraging habitat. Central place foraging theory predicts that animals will preferentially use space in close proximity to high-quality foraging areas [47]. However, the importance of proximity to foraging areas in roost-site selection for a volant species such as the Indiana bat is not well understood [16]. Multiple studies have shown that Indiana bats tend to roost near water sources that are generally regarded as optimal foraging areas [36,48,49,50], but that they also can move relatively long distances away from foraging areas to roost during the day [45,51] and are known to day-roost in upland habitat types [25,45,52,53]. We evaluated the prediction that Indiana bats would select day-roosts in close proximity to high-quality foraging areas, extracting the predicted foraging value for each used and available location calculated from a companion study on Indiana bat foraging ecology that occurred during the same time period [29]. Essentially, nighttime radio-tracking data of Indiana bats during this period of study revealed a strong selection for foraging in areas in close proximity to water and forest edge (see [29] for further details).

#### Tree scale

Multiple factors are hypothesized to influence roost-site selection at the scale of the individual tree, including tree species, condition, DBH, and height [16,27,28,54]. We hypothesized that Indiana bats would select roosts in large-diameter bitternut hickory, sugar maple and American elm over other tree species (note *Populus* spp. was lumped into “other” for this level of analysis due to small sample size) at Fort Drum. The decay condition of a tree or snag is noted as an important factor in roost-site selection, where trees in advanced decay stages are more likely to have loose bark or crevices for roosting [16,17,37]. Therefore, we hypothesized that bats would select for trees or snags in advancing states of decay (although not to the point whereby no bark remained on a tree). The height of an individual roost tree in relation to the surrounding canopy can influence roost-site selection due to the thermoregulatory benefits of solar exposure of roost sites [16,25,28,55], such that dominant trees (or formerly dominant in the case of snags) within the canopy might provide warmer roost locations. Therefore, we hypothesized that bats would select for trees that maintained a dominant or co-dominant crown position.

Within the 11.4-radius plot surrounding each identified roost tree, we measured and recorded the species, decay class, DBH and crown position of all trees using methods outlined above (Fig 1). All plot site measurements were recorded within the same month the individual roost tree measurements were recorded (with the exception of a small number of 2007 roost tree plots that were recorded in 2008).

#### Sex-linked factors

Although few comparative studies exist, previous research of individual male and female Indiana bats suggest similar patterns of selection at the scale of the roost-tree [17,42,52]. However, given the burden of rearing young during the summer maternity season, we hypothesized that male and female Indiana bats plausibly would exhibit different roost-site selection at the stand and landscape scales. First, thermoregulation is critical for enhancing fetal development and juvenile growth during the summer maternity season [56]. Therefore, we hypothesized that female Indiana bats might be more likely to select resources based on topographic positioning (i.e., slope and aspect) at the landscape scale. Second, to protect young, we hypothesized that females would select sites farther from human development at the landscape scale [16]. Third, because of increased energetic needs of females rearing offspring [57], we hypothesized that they would be more likely to select roosts closer to high-quality foraging areas at the stand scale. Finally, because female bats could select roost sites differently during the summer maternity season (May 15-August 15) compared to when volant young are post-weaning in the fall (August 16-October 15), we accounted for an effect of season in our analyses.

### Resource selection analyses

We modeled roost-site selection with hierarchical Bayesian discrete choice models [58,59]. The observational units in this study were the choice set (i.e., the used roost tree and the available alternative choices associated with each used tree). We assumed the roost tree selected from choice set *i* by bat *j* was a multinomial random variable:

$$y_{ij}∼\text{Multinomial}(\pi_{ij},1),$$

where **y**_*ij*_ is a *K*_*ij*_-dimensional vector of 0s (indicating available alternatives) and 1 (indicating the used roost tree), *K*_*ij*_ is the number of used and available alternatives in choice set *i*, and **π**_*ij*_ is a *K*_*ij*_-dimensional vector denoting the probability of selecting any of the alternatives in choice set $i\left(\sum {}_{k=1}^{K_{i}}\pi_{ijk}=1\right)$. We assumed a latent “utility” associated with each used and available alternative:

$${\text{u}}_{\text{ijk}}=\text{x}{'}_{\text{ijk}}\beta_{\text{j}}+\varepsilon_{ijk},$$

where **x**_*ijk*_ is an *L*-dimensional vector of variables associated with alternative *k*, **β**_*j*_ is a conformable vector of slope parameters unique to bat *j*, and *ε*_*ijk*_ ~ Normal(0, *σ*_*ε*_^2^) is an alternative-specific random effect. We assume random slope coefficients for each bat by modeling each element *l* = 1, …, *L* of **β**_*j*_ hierarchically:

$$\beta_{jl}∼\text{Normal}(\mu_{\text{jl}},\sigma_{l}^{2}),$$

where *μ*_*jl*_ and *σ*_*l*_^2^ are the population-level mean and variance, respectively, of slope coefficients associated with variable *l*. Note that assuming random slope coefficients for each bat appropriately accounts for repeated and unequal observations from each bat [59]. We model the population-level mean of slope coefficient *l* as:

$$\mu_{l}=S\Theta_{l},$$

where **S** is a *J* × *M* design matrix, *J* indicates the total number of bats observed, *M* indicates the number of variables in the population-level mean model (see below), and **θ**_*l*_ is a conformable vector of parameters. We calculated the probability bat *j* selected alternative *k* within choice set *i* as a function of the latent utility:

$$\pi_{ijk}=\frac{\text{exp}({\text{u}}_{\text{ijk}})}{\sum_{a=1}^{K_{i}}\text{exp}({\text{u}}_{ija})}\;.$$

We assumed the following prior distributions:

$$\begin{array}{l} \Theta_{l}∼\text{Multivariate Normal}(0,\text{I}),\\ \sigma_{l}^{2}∼\text{Inverse Gamma}(1,1),\\ \sigma_{\varepsilon }^{2}∼\text{Inverse Gamma}(1,1). \end{array}$$

We used model selection techniques to evaluate evidence for sex and season-specific differences in random slope coefficients. At each scale, we fit 5 models assuming **μ**_*l*_ is a function of 1) sex, season, and their interaction (i.e., *M* = 4); 2) sex and season (i.e., *M* = 3); 3) sex only (i.e., *M* = 2); 4) season only (i.e., *M* = 2); and 5) neither sex nor season (i.e., *M* = 1, constant **μ**_*l*_). We ranked each model with Watanabe-Akaike Information Criterion (WAIC), a fully Bayesian information criterion analogous to Akaike’s Information Criterion [60,61] and report results from the top-ranked model at each scale. We base inference on the population-level means associated with the top-ranked model at each scale.

We evaluated goodness of fit of the top-ranked model at each scale with posterior predictive checks [60,62]. Briefly, we calculated the deviance function [63] from observed data (*T*_*y*_) and from data simulated assuming the top-ranked model was the data-generating model (*T*_*rep*_). We then calculated a Bayesian *p*-value *p*_*B*_ = Pr(*T*_*rep*_ > *T*_*y*_) from posterior simulations and assumed reasonable fit if 0.1 < *p*_*B*_ < 0.9.

We conducted posterior sampling with Stan v. 2.6.0 [64], implemented in R v. 3.2.1 [65] via the RStan v. 2.6.0 interface [66]. We simulated 3 posterior chains for each model, running each chain until adequate convergence was achieved ($\hat{R}$ ≤ 1.1, [60]). We thus ran each chain for 11,000 iterations for the landscape-level models and 21,000 iterations for both the stand and tree-level models. We discarded the 1^st^ 1,000 iterations as warm-up and thinned the remaining chains at such a rate that we kept 1,000 posterior samples from each chain (3,000 posterior samples total).

## Results

During 2007–2011, we radiotracked 33 Indiana bats (10 males, 23 females) within our study area. We located 348 roost events, many of which involved sites that were used by individuals repeatedly or by multiple bats simultaneously (Table 1). In total, we observed bats using 116 unique roost trees over the course of our study (Table 2).

**Table 1:** Indiana bats (n = 33) radiotracked to different day-roost sites during summer months (May–October) between 2007–2011 at Fort Drum Military Installation, New York, USA. Note: some roosts were used by multiple bats.

Bat ID	Age	Sex	Year	Month	Nights tracked	# different roosts
150–003	A	M	2008	May/June	21	5
150–019	A	F	2008	May	21	8
150–041	A	F	2008	July	16	4
150–059	A	F	2008	July	11	7
150–091	A	M	2008	June	1	1
150–128	A	F	2008	July	12	5
150–147	J	M	2008	August	17	3
150–222	J	F	2008	Sept	13	9
150–282	A	F	2009	June	12	7
150–300	A	F	2009	June	6	3
150–316	A	F	2009	July	6	4
150–425	A	F	2007	July	8	3
150–459	J	M	2009	August	14	8
150–485	A	M	2008	Sept	13	8
150–517	J	F	2010	August	9	4
150–567	J	F	2008	August	4	1
150–603	A	F	2008	July	5	5
150–887	A	F	2007	June	8	4
150–952	A	F	2007	June	5	4
150–968	A	F	2007	July/August	6	3
150–990	A	F	2007	June	4	3
151–014	A	F	2007	July	5	4
151–036	A	F	2007	July	8	1
151–058	J	F	2007	August	15	4
151–084	A	F	2007	July	7	2
151–102	J	F	2007	August	12	3
151–125	A	M	2007	Sept/Oct	21	14
151–158	A	F	2007	Sept/Oct	19	12
151–200	J	F	2007	Sept/Oct	18	6
151–852	A	M	2010	August	6	4
172–521	A	F	2008	June/July	9	4
172–462	A	M	2008	June/July	9	4
172–2591	A	F	2011	July	7	2

**Table 2:** Characteristics of 116 roost trees selected by Indiana bats at Fort Drum Military Instillation, New York, USA between 2007–2011. Diameter at breast height and decay class are presented as averages, with standard error values in parentheses, and crown positions are listed as tallied occurrences in each of 4 possible crown classes. Decay class values are on a scale of 1 to 8, with 1 being a healthy intact tree, 4 being a dead snag with loose bark, and 8 being a residual stump [32,33,34].

				Crown position			
Tree species	n	Diameter at breast height (cm)	Decay	Dominant	Co-dominant	Intermediate	Suppressed
American elm *(Ulmus* *americana)*	58	32.58 (1.77)	4.48 (0.11)	23	27	4	4
Sugar maple *(Acer saccharum) *	20	46.10 (6.43)	3.95 (0.36)	5	7	7	1
Bitternut hickory *(Carya cordiformis)*	10	29.31 (2.35)	5.10 (0.62)	0	2	3	5
Eastern white pine *(Pinus strobus)*	6	34.90 (5.88)	5.17 (0.70)	1	2	2	1
Red maple *(Acer rubrum)*	5	49.99 (13.04)	4.40 (0.40)	4	1	0	0
Quaking aspen *(Populus tremuloides)*	5	32.83 (2.21)	4.60 (0.60)	4	0	0	1
Black cherry *(Prunus serotina)*	5	36.36 (4.05)	2.60 (0.60)	1	2	1	1
American beech *(Fagus grandifolia)*	3	42.67 (12.10)	6.00 (1.00)	1	0	0	2
Scots pine *(Pinus sylvestris)*	2	31.12 (3.94)	5.50 (0.50)	0	0	2	0
Silver maple *(Acer saccharinum)*	1	48.26 (-)	5.00 (-)	1	0	0	0
Butternut (*Juglans cinerea*)	1	72.39 (-)	2.00 (-)	1	0	0	0

### Landscape scale

We modeled landscape-level resource selection from 348 choice sets, each of which included *K* = 6 alternatives. We obtained *p*_*B*_ = 0.58 from the top-ranked model, suggesting reasonable goodness-of-fit.

**Fig 2:**
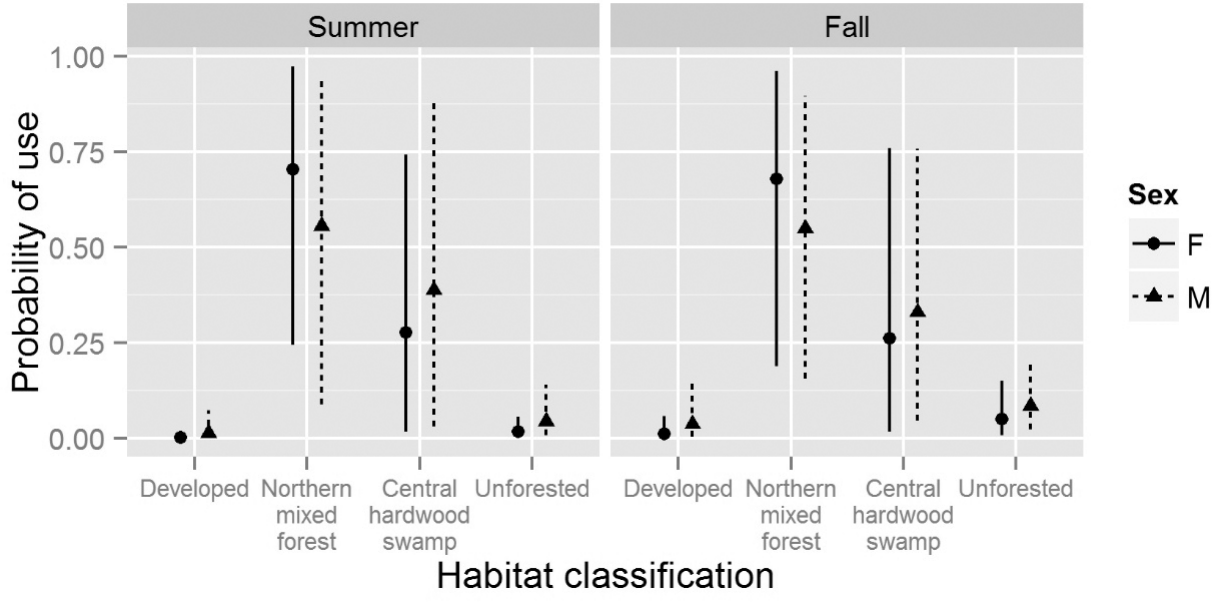
Predicted probability of selecting a roost as a function of habitat classification for male and female Indiana bats (*Myotis sodalis*) in summer and fall months in Fort Drum, NY, USA between 2007–2011. Points indicate mean posterior probability of use and vertical lines represent the limits of 95% credible intervals. This Fig assumes bats are faced with a choice of 4 potential roosts, one in each habitat classification. All other variables are assumed constant across habitat classifications.

The top-ranked landscape-level model suggested individual-level slope coefficients varied by sex, season, and their interaction (Table 3). We found evidence that bats are most likely to select roosts located in northern mixed forests, regardless of sex or season (Fig 2). There was enough variation in individual-level slope coefficients among sexes and seasons that there was no discernable population-level influence of distance from development, aspect, and slope on the probability a bat would select a roost (i.e., 95% credible intervals of all elements of **θ**_*l*_ overlapped 0 for each of these variables).

**Table 3:** Model selection results evaluating the evidence for sex- and season-specific differences in resource selection of Indiana Bat (*Myotis sodalis*) in Fort Drum, NY, USA between 2007–2011. Lower WAIC is better.

Scale	Model	WAIC
Landscape	Sex, Season, Sex × Season	614.14
	Sex, Season	617.52
	Sex	628.82
	Season	629.03
	Constant	634.17
Stand	Sex, Season, Sex × Season	434.83
	Sex, Season	442.13
	Season	442.67
	Sex	444.29
	Constant	448.19
Tree	Constant	331.11
	Season	332.18
	Sex	332.22
	Sex, Season	333.15
	Sex, Season, Sex × Season	333.55

### Stand scale

We modeled stand-level resource selection from 314 choice sets, each of which included *K* = 5 or *K* = 6 alternatives. We obtained *p*_*B*_ = 0.55 from the top-ranked model, suggesting reasonable goodness-of-fit.

**Fig 3:**
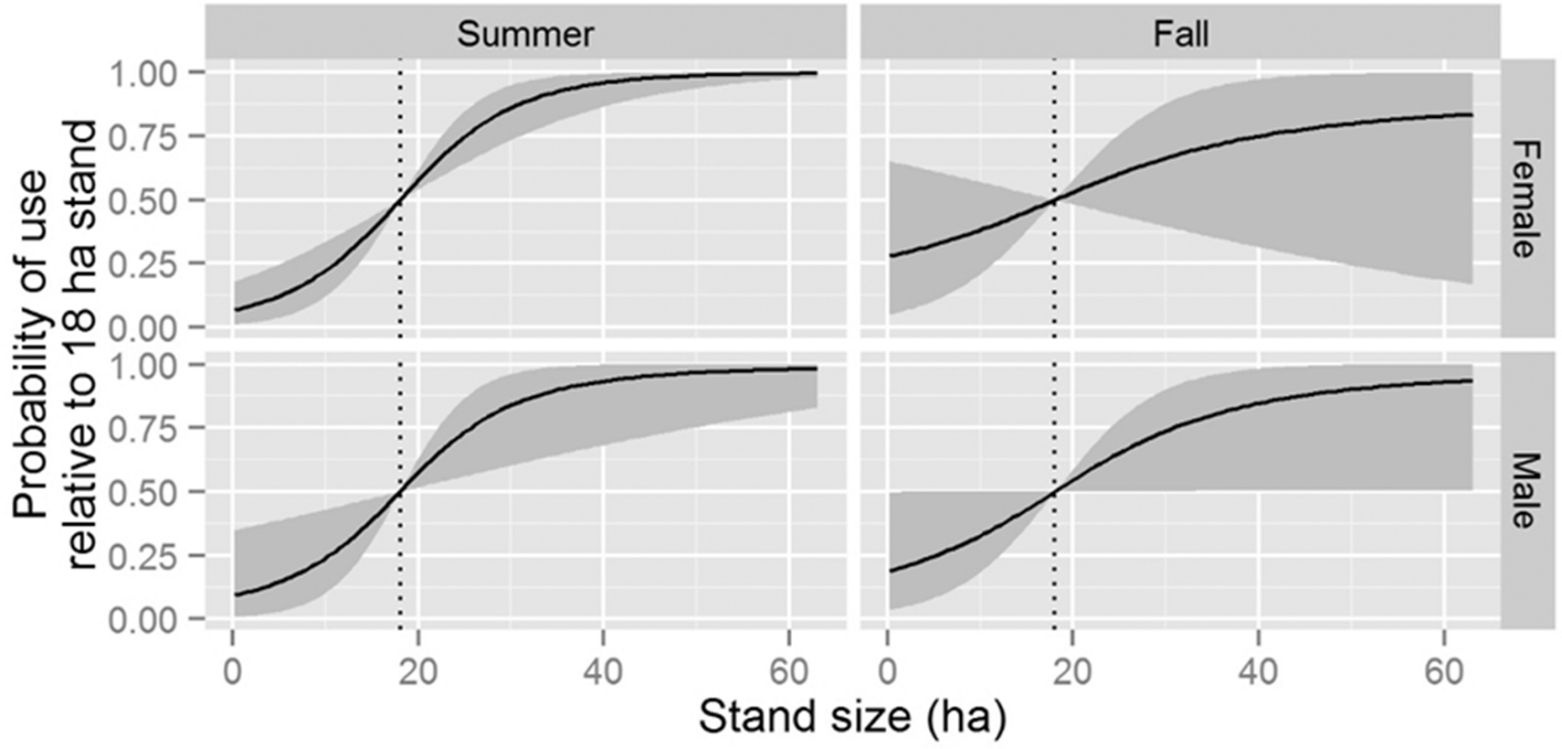
Predicted probability of selecting a roost as a function of stand size for male and female Indiana bats (*Myotis sodalis*) in summer and fall months in Fort Drum, NY, USA between 2007–2011. Solid lines indicate mean posterior probability of use and gray ribbons represent the limits of 95% credible intervals. Each panel assumes bats are faced with a choice of 2 potential roosts: one fixed at the observed mean stand size (vertical dashed line) and the other represented by the value of the *x*-axis. All other variables are assumed constant.

**Fig 4:**
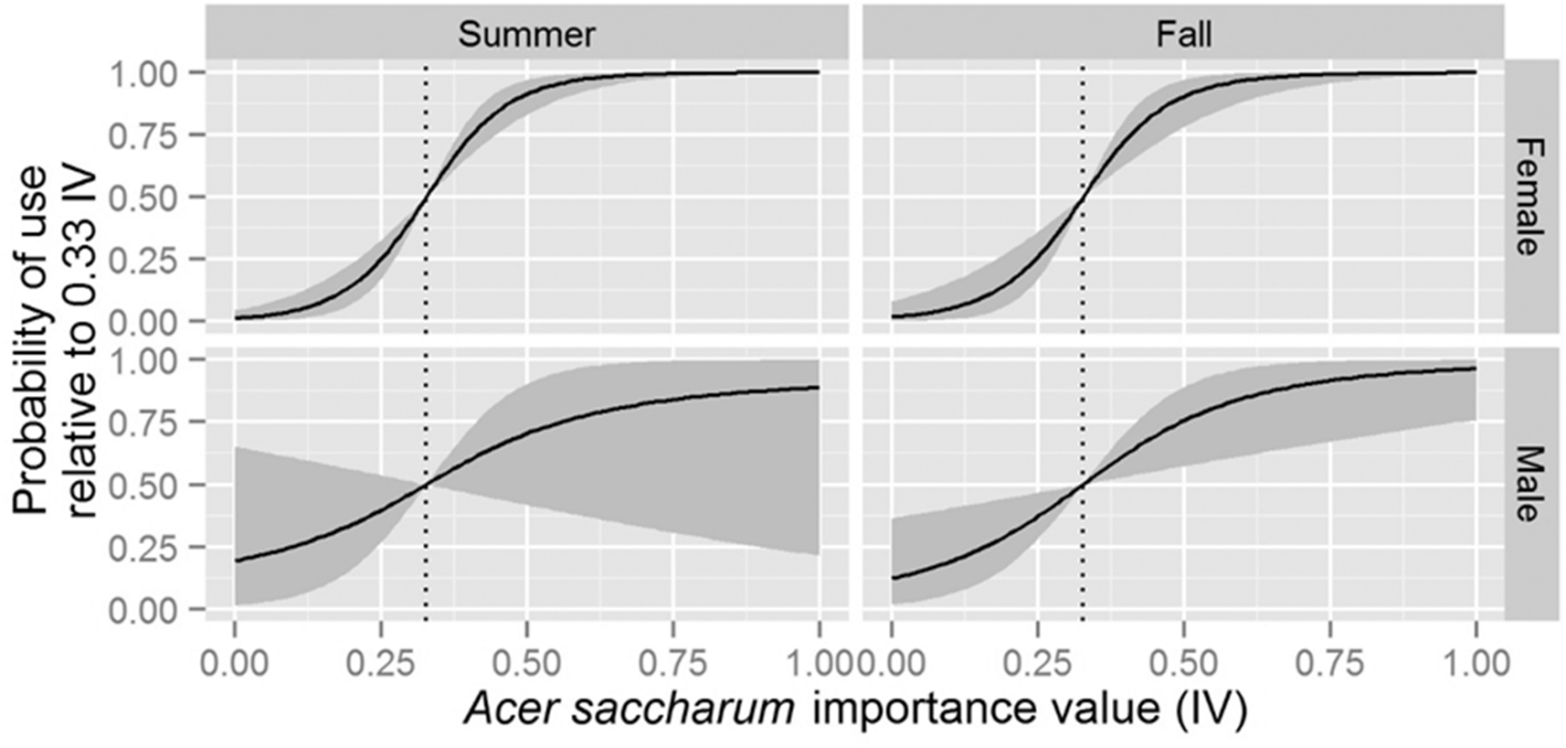
Predicted probability of selecting a roost as a function of *Acer saccharum* importance value for male and female Indiana bats (*Myotis sodalis*) in summer and fall months in Fort Drum, NY, USA between 2007–2011. Solid lines indicate mean posterior probability of use and gray ribbons represent the limits of 95% credible intervals. Each panel assumes bats are faced with a choice of 2 potential roosts: one fixed at the observed mean *Acer saccharum* importance value (vertical dashed line) and the other represented by the value of the *x*-axis. All other variables are assumed constant.

The top-ranked stand-level model suggested individual-level slope coefficients varied by sex, season, and their interaction (Table 3). We found evidence that both males and females selected roosts in stands of greater total area in the summer months, but that only males selected these larger stands in the fall months (Fig 3). We also found evidence that both males and females selected roosts within stands with high sugar maple importance value in the fall months, but that only females selected roosts within stands with high sugar maple importance value in the summer months (Fig 4). There was enough variation in individual-level slope coefficients among sexes and seasons that there was no discernable population-level influence from basal area; average tree diameter; American elm, hickory (*Carya* spp) or *Populus* spp. importance value; and predicted foraging value (i.e., 95% credible intervals of all elements of **θ**_*l*_ overlapped 0 for each of these variables).

### Tree scale

We modeled tree-level resource selection from 131 choice sets, each of which included from *K* = 6 to *K* = 56 alternatives. We obtained *p*_*B*_ = 0.66 from the top-ranked model, suggesting reasonable goodness-of-fit.

**Fig 5:**
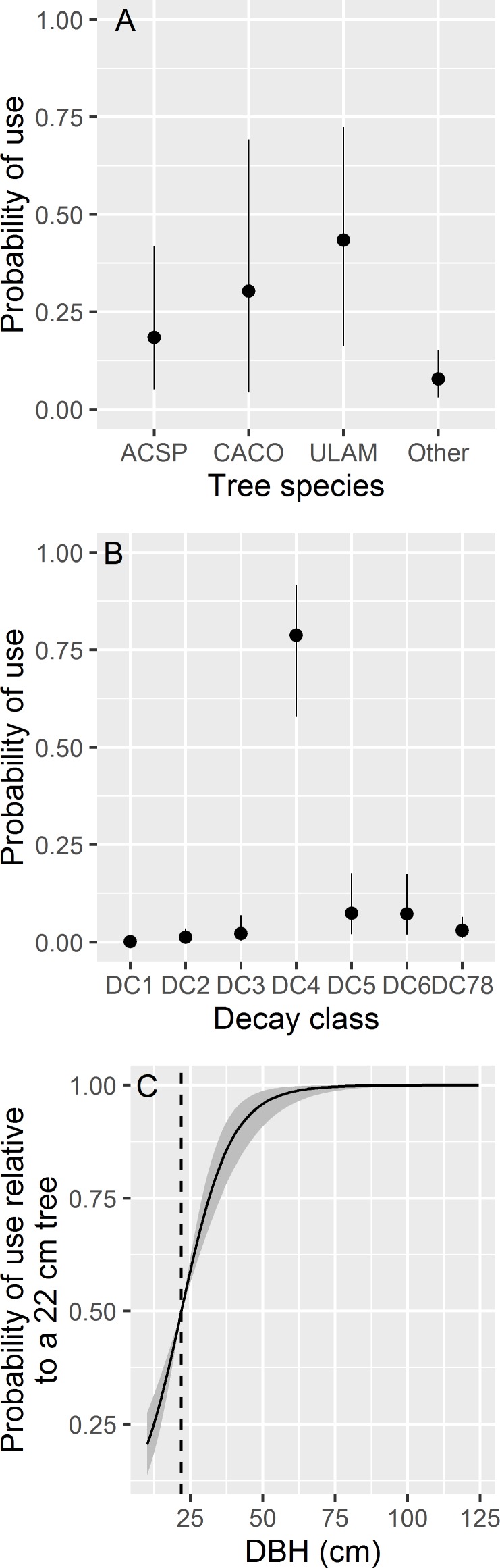
Predicted probability of roost tree selection for Indiana bats (*Myotis sodalis*) in Fort Drum, NY, USA between 2007–2011. In panel A), points indicate mean posterior probability of use and vertical lines represent the limits of 95% credible intervals. This panel assumes bats are faced with a choice of 4 potential roosts: one in *Acer* species (ACSP), one in *Carya cordiformis* (CACO), one in *Ulmus americana* (ULAM), and one that is any other possible tree species (Other). In panel B), points indicate mean posterior probability of use and vertical lines represent the limits of 95% credible intervals. This panel assumes bats are faced with 7 potential roosts, classified from decay class 1 (DC1) through decay class 7 or 8 (DC78). In panel C), the solid line indicates the mean posterior probability of use and gray ribbons represent the limits of 95% credible intervals. This panel assumes bats are faced with a choice of 2 potential roosts: one fixed at the observed mean diameter at breast height (DBH, vertical dashed line) and the other represented by the value of the *x*-axis. All other variables are assumed constant in all panels.

The top-ranked tree-level model suggested each individual-level slope coefficient was reasonably represented by a constant mean across sexes and seasons (Table 3). We found bats were most likely to select roosts in American elm (Fig 5A); to avoid roosts in trees classified with decay class 1 and to select roosts in classified as decay class 4 (Fig 5B); and to select roosts in trees with large DBH (Fig 5C). Despite 82 of 116 roost trees being either dominant or co-dominant in crown position, we found no discernable population-level influence from crown position on roost-tree selection (i.e., 95% credible intervals of all elements of **θ**_*l*_ overlapped 0 for each of these variables).

## Discussion

Indiana bats on Fort Drum selected roost sites based on environmental conditions at multiple spatial scales, which further emphasizes the need for additional multi-scale investigations into bat roost-site selection. Previously, most research and management emphasis has been focused on management and retention of individual roost trees [12,13,15,16]. However, it is increasingly apparent that for many temperate-zone bat species, identifying patterns in roost-site selection is as much or more of a function of forest stand and landscape-scale factors than of individual roost tree characteristics [10,37,67,68]. Our ability to predict roost-site selection was better at the larger stand and landscape scales than at the individual tree scale–a scale that itself was highly variable. Therefore, due to the plasticity often exhibited by Indiana bats in their selection of individual roost trees, further investigations are needed to support the design of forest management practices around multi-scale habitat conditions.

The predicted selection of northern mixed-hardwood forest at Fort Drum by Indiana bats corresponds largely to previous research on bat roost ecology of the species throughout much of the eastern United States [16,17,42]. Although considerable focus has centered on retaining specific roost trees within managed forests, surprisingly little is known about how forest stand management practices could influence Indiana bat roost ecology [16]. We found that the area, structure and composition of forest stands were important predictors of roost-site selection. Selection of roost sites in stands with high basal area values is likely a result of a general trend for bats to either select for stands with mature, large-diameter trees in varying states of decay, or stands with crowded mid-sized stems with snags present as roost sites. The selection of American elm appears fairly unique to the forest conditions found at Fort Drum in this portion of Indiana bat distribution. However, the selection of stands with a high maple component, and of day-roosts in sugar and red maple, is consistent with previous findings from the region that document use of maples as roost sites by both male [17] and female Indiana bats [42]. By comparison, Indiana bats are known to use roost sites in eastern hemlock-hardwood stands in the southern Appalachians [27] and Central Hardwood forests and swamp edge habitat in much of the Midwest [36,69], with some use of silver maple (*Acer saccharinum*) and green ash (*Fraxinus pennsylvanica*) associated swamp forests in the northern Midwest [70]. Collectively, it is evident that Indiana bats exhibit plasticity in their selection of species composition of forest stands within which to establish roosts depending on site conditions and regional characteristics. Nonetheless, they generally use forests with similar structural composition, i.e., large-diameter trees with sloughing bark, crevices or cavities. Evidence of use of maple-dominated stands by Indiana bats in the Northeast is particularly important and encouraging given the increasingly common occurrence of maple species, particularly red maple, in the region resulting from past and current harvesting practices and from the mesophication phenomenon. The latter of which is a result of forest fire suppression that has encouraged a shift to mesic, shade-tolerant tree species in current and presumably future forests [71,72].

Although there was a general trend for Indiana bats on Fort Drum to select larger forest stands with a higher sugar maple component, we did observe subtle sex-specific, seasonal differences that could be of conservation interest. In general, a trend toward selection of larger stands by Indiana bats on Fort Drum was likely influenced by historical forest management practices, whereby larger forest patches were typically left in areas that were too steep, wet or rocky for agriculture or harvesting in the past [73]. Further, because some larger forest patches in the region often were maintained for firewood and maple syrup production, these stands typically contained a greater abundance of residual shade-tolerant maples [74]. These trees and their progeny were able, therefore, to mature into larger diameter classes and/or develop into stands with some level of decadence, subsequently becoming potential Indiana bat roosts. Therefore, our observed trend of female bats being more likely to select stands with a high maple component during the summer months is likely in response to the availability of these large diameter trees as maternity roost sites. It is unclear why only males selected larger stands during fall months, but could suggest that females in the fall are no longer restricted to maternity roosting areas in large forest stands and take advantage of foraging opportunities near forest edges [29], and thus willing to day-roost in smaller forest fragments.

Selection of individual roost trees was highly variable on Fort Drum, but similar to studies in other regions, Indiana bats tended to select trees that were larger and in moderate to advanced decay classes [16,27,50]. However, failure to find bats strongly selecting roost trees with dominant or co-dominant crown positions suggests that large trees with suitable solar exposure for roosting were likely not limited and roosts were selected more in response to exfoliating bark or roost crevice availability. The selection of maples as roost trees also is consistent with previous research [16,44,45]. However, at the roost site level at Fort Drum, Indiana bats selected individual, large-diameter American elm trees, which is somewhat unique compared to other portions of the Indiana bat’s distribution. Because of Dutch Elm Disease (*Ophiostoma novo-ulmi*), most American elm-dominated stands remaining locally provide few roosting opportunities, as these stands are often characterized by dense, small-bole structured trees that typically fail to reach large diameters before die-back. Conservation of American elm presents a silvicultural challenge as tree mortality from Dutch elm disease continues to remove older trees at Fort Drum at rates higher than pre-disease background, offering little chance of even partial replacement of boles with structure and size sufficient for Indiana bat use. Although previous studies have shown that Indiana bats select hickories with exfoliating bark [16], hickories with tight bark that develop cracks and crevices such as bitternut hickory also are used as roost sites [25]. However, bitternut hickory was not as consistently selected at Fort Drum, perhaps due to its relatively patchy distribution and lack of fine-scale suitable roosts, i.e., broken tops or bole cracks. Conversely, research conducted approximately 250 km east of Fort Drum in the Champlain Valley showed that the primary trees selected for roosting by Indiana bats were shagbark hickory (*Carya ovata*) and black locust (*Robinia pseudoacacia*) with exfoliating bark and high snag longevity, respectively [42,54]. Therefore, where detailed information on specific roost trees selected by Indiana bats is available, it may be possible focus on conservation of individual trees or tree species. However, where such information is lacking, collectively it is evident that the prioritization of stand-scale availability of large-diameter trees or snags showing advanced states of decadence is important [10], as well as the retention of appropriate tree species within stands to have sustainable roost replacement into the future.

In addition to forest characteristics, topographic features can likely have complex, often sex- and site-specific effects on Indiana bat roost-site selection. Aspect has previously been shown to be a consistent predictor of female Indiana bat roost-site selection in the Champlain Valley in Vermont [42] and of male roost-site selection in the high Allegheny Mountains to the south in West Virginia [17]. However, despite the likely thermoregulatory benefits for Indiana bats roosting on south-facing aspects [17], we observed no strong effect of aspect on roost-site selection by Indiana bats of either sex. However, in contrast to Watrous et al. [42], we observed that female Indiana bats selected roosts at increasing percent slope. This suggests that female bats on Fort Drum likely selected roost trees that had more solar exposure on upper slopes and ridgelines where thermoregulatory benefits could be maximized [16]. Alternatively, because steeper slopes were more difficult to harvest historically [73], it is simply possible that these areas retained uncut trees or large residual ones following selective harvests that then developed into trees suitable for roosts. Regardless, differences between studies are likely to be due, in part, to overall terrain (though elevations overall were similar). The Champlain Valley was composed of distinct ridge and valley topography (where south facing aspect likely maximized solar exposure) compared to lower elevation and more rolling hills of Fort Drum (where increased slope likely maximized solar exposure). Similar to the observations of Johnson et al. [17], showing both positive and negative effects of percent slope on male Indiana bat roost selection, our failure to observe a strong positive relationship with slope for males suggests greater plasticity in roost-site selection during summer. Overall, these contrasting effects of topography on Indiana bat roost-site selection are likely a result of sex-specific thermoregulatory demands, general terrain of an area, and interactions with site-specific forest stand conditions and water/foraging area availability.

In contrast to our original hypothesis, roost-site selection did not follow central place foraging theory [47], where bats would be predicted to roost in areas close to high-quality foraging sites. These findings were likely due to two site-specific factors. First, we found that roost site selection was positively associated with large forest stands (Fig 3), but high-quality foraging areas were not located within large forest stands but rather near forest edges adjacent to marshes, streams, or ponds [29]. Second, foraging areas were widely distributed across our study area [29]. Therefore, because Indiana bats often do move long distances between foraging and roosting areas [45,51], foraging areas were not likely to have been a limiting resource in our study system that would lead to restricted roosting behavior.

Differences between foraging and roosting habitat, and the need for bats to move between the two, suggest that the availability and juxtaposition of larger forest stands with large trees or snags and wetland edges has important ecological implications for Indiana bats. Our multi-year study was initiated immediately prior to the arrival of White-nose Syndrome (2007), and continued in subsequent years (2008–2011) when rapid declines in populations of Indiana bats and other bat species were observed [18,20]. Should White-nose Syndrome threats be mitigated, high-quality foraging sites and secure roost areas during the summer maternity season will be important for enhancing population recruitment and overall recovery. This will require an integration of existing knowledge of Indiana bat foraging and roosting behavior, which can involve spatially distinct, yet interrelated, periods of its life history. In practice, these approaches will need to consider land management and development in the context of knowledge about important site-specific roosting and foraging areas if adverse impacts on bats are to be minimized. For example, at Fort Drum and the surrounding local area, monitoring of the fate and status of sugar maple and large-diameter American elm as substantial forest components and their associated day-roost value to Indiana bats in the near- and long-term is paramount. Where such site-specific information is not available, our study illustrates how a multi-scale spatial framework can be used to evaluate and identify important roosting habitats, and address potentially complex regulatory requirements for this endangered species.
