# Correction: Seeing the Forest through the Trees: Considering Roost-Site Selection at Multiple Spatial Scales

**DOI:** 10.1371/journal.pone.0169815

**Published:** 2017-01-03

**Authors:** David S. Jachowski, Christopher T. Rota, Christopher A. Dobony, W. Mark Ford, John W. Edwards

There are errors in the Abstract. The correct Abstract is: Conservation of bat species is one of the most daunting wildlife conservation challenges in North America, requiring detailed knowledge about their ecology to guide conservation efforts. Outside of the hibernating season, bats in temperate forest environments spend their diurnal time in day-roosts. In addition to simple shelter, summer roost availability is as critical as maternity sites and maintaining social group contact. To date, a major focus of bat conservation has concentrated on conserving individual roost sites, with comparatively less focus on the role that broader habitat conditions contribute towards roost-site selection. We evaluated roost-site selection by a northern population of federally-endangered Indiana bats (Myotis sodalis) at Fort Drum Military Installation in New York, USA at three different spatial scales: landscape, forest stand, and individual tree level. During 2007–2011, we radiotracked 33 Indiana bats (10 males, 23 females) and located 348 roosting events in 116 unique roost trees. At the landscape scale, bat roost-site selection was positively associated with northern mixed forest. At the stand scale, we observed subtle differences in roost site selection based on sex and season, but roost selection was generally positively associated with larger stands, and a greater sugar maple (Acer saccharum) component. We observed no distinct trends of roosts being near high-quality foraging areas of water and forest edges. At the tree scale, roosts were typically in American elm (Ulmus americana) or sugar maple of large diameter (>30 cm) of moderate decay with loose bark. Collectively, our results highlight the importance of considering day roost needs simultaneously across multiple spatial scales. Size and decay class of individual roosts are key ecological attributes for the Indiana bat, however, larger-scale stand components that are products of past and current land use interacting with environmental aspects such as landform also are important factors influencing roost-tree selection patterns.

There are errors in the penultimate sentence of the first paragraph of the Discussion. The correct sentence is: Our ability to predict roost-site selection is not better or worse at any of the scales we examined.

There are errors in the second paragraph of the Discussion. The following sentence should be removed: Selection of roost sites in stands with high basal area values is likely a result of a general trend for bats to either select for stands with mature, large-diameter trees in varying states of decay, or stands with crowded mid-sized stems with snags present as roost sites.

There is an error in the third sentence of the second paragraph of the Discussion. The correct sentence is: We found that the area and composition of forest stands were important predictors of roost-site selection.

There are errors in the fifth paragraph of the Discussion. The fifth paragraph should read: In addition to forest characteristics, topographic features can likely have complex, often sex- and site-specific effects on Indiana bat roost-site selection. Aspect has previously been shown to be a consistent predictor of female Indiana bat roost-site selection in the Champlain Valley in Vermont [42] and of male roost-site selection in the high Allegheny Mountains to the south in West Virginia [17]. However, despite the likely thermoregulatory benefits for Indiana bats roosting on south-facing aspects [17], we observed no strong effect of aspect on roost-site selection by Indiana bats of either sex.
